# Study on identification and analysis of biomarkers related to neutrophils extracellular traps in childhood asthma

**DOI:** 10.1097/MD.0000000000043489

**Published:** 2025-08-01

**Authors:** Yuexuan Wu, Wen Zhao, Yalong Yang, Jinhai Ma

**Affiliations:** a Department of Pediatrics, General Hospital of Ningxia Medical University, Yinchuan, China; b Genetics and Reproductive Medicine, School of Basic Medical Sciences, Ningxia Medical University, Yinchuan, China; c Department of Pediatrics, General Hospital of Ningxia Medical University, Yinchuan, China.

**Keywords:** biomarkers, childhood asthma, immune cells, machine learning, neutrophil extracellular traps

## Abstract

Childhood asthma (CA) is a prevalent chronic inflammatory disease affecting the respiratory system, with neutrophil extracellular traps (NETs) playing a key role in triggering CA. Therefore, identifying NET-related biomarkers for CA treatment is crucial. In this study, transcriptome data were utilized to identify differentially expressed genes (DEGs) associated with CA. Weighted gene co-expression network analysis was performed to identify module genes correlated with NET-related gene scores. Candidate genes were obtained by intersecting the DEGs and key module genes. Advanced machine learning techniques were then applied to these candidates to identify potential biomarkers. Subsequently, immune infiltration and gene set enrichment analyses were conducted based on these biomarkers. Finally, the expression levels of the identified diagnostic biomarkers were analyzed at the transcriptional level. A total of 34 DEGs related to CA were identified, followed by the identification of 2611 module genes associated with NET-related gene scores. Eleven candidate genes were selected for further analysis using a Venn diagram. Machine learning techniques helped identify 4 key biomarkers linked to NETs: FCGR2B, FCRL5, CCR2, and FCRL1. Furthermore, 5 immune cells were found to be differentially infiltrated into the immune microenvironment of CA. All identified biomarkers were associated with the “other glycan degradation” pathways, and notably, these biomarkers exhibited significantly higher expression in the CA group compared to the control group. In conclusion, 4 NET-related biomarkers (FCGR2B, FCRL5, CCR2, and FCRL1) linked to CA were identified, providing a theoretical basis for the development of treatments for CA.

## 1. Introduction

Asthma is a prevalent chronic inflammatory disease that affects the respiratory system on a global scale,^[1]^ both in developing and developed countries.^[2,3]^ The etiology of asthma involves multiple factors, but the specific mechanisms are not yet clear.^[4]^ This disease exhibits genetic heterogeneity,^[5]^ and can be caused by genetic factors, environmental pollution, lifestyle choices, and other influences that can trigger inflammation associated with airway hyperreactivity and variable airflow obstruction,^[5]^ some patients may also have rashes and other allergic conditions.^[6]^ Asthma severely affects not only the health and daily lives of adults but also the physical and mental development of children, posing serious challenges.^[1,7]^

The annual morbidity of childhood asthma (CA) has increased with air pollution.^[8]^ The long-term use of anti-asthma medications may lead to reversal and resistance, so we need to find a more effective method to improve the symptoms of asthma. A lack of efficient treatment would lead to drug resistance and respiratory dysfunction in the long run.^[9,10]^

Neutrophils are one of the most important immune cells in the immune system and play a key role in many diseases, especially in inflammation and immunity.^[11]^ Neutrophil extracellular traps (NETs) are composed of granular proteins, histones, and extracellular DNA, which can degrade virulence and kill bacteria.^[12–16]^ After nearly 15 years of research, it has been found that NETs are not only resistant to infection but also play an important role in noninfectious inflammation, such as autoimmunity, blood coagulation, and acute lung injury.^[17]^ Recent research indicates that NETs play a role in advancing inflammation associated with asthma.^[18]^ Some models have elucidated the effects of NETs because of extracellular histones and tried to clarify the mechanism. Han et al initially showed that NETs can cause damage to human airway epithelial cells in *vitro* using a mouse model of asthma.^[19]^ In addition, several lines of evidence indicate a relationship between NETs and respiratory tract diseases, which induce an immune inflammatory response by secreting immune factors from neutrophils. However, there has been less research on NETs in CA than in adult patients and animal models.^[18]^

Based on the relevant datasets of CA in the public database, this study revealed the diagnostic significance of NET-related genes in CA by means of bioinformatics, and explored its potential molecular regulation mechanism in CA to offer a theoretical basis for the development of novel therapeutic approaches and medications in clinical settings.

## 2. Materials and methods

### 2.1. Data acquisition

The gene expression profile of the GSE40888 dataset (training set) was obtained from the Gene Expression Omnibus database (http://www.ncbi.nlm.nih.gov/geo/), which included 40 normal and 24 CA samples. The GSE40732 dataset consisted of 97 CA and 97 normal samples, which were considered for the validation set. All samples were collected from peripheral blood mononuclear cells. A sum of 170 neutrophil extracellular trap-related genes (NETRGs) were derived from previous research.^[15]^

### 2.2. Analysis of differential genes

The “limma” package^[20]^ was utilized to analyze and identify genes that were expressed differently between the normal and CA groups in the GSE40888 dataset, applying thresholds of *P*.adj < .05 and |log_2_FC| > 0.5 for significant variation and being defined as DEGs.

### 2.3. Weighted gene co-expression network analysis

For the same dataset, GSE40888, the “GSVA” package was employed to compute the single-sample gene set enrichment analysis (ssGSEA) scores for NETRGs across all samples. These ssGSEA scores were then used as clinical traits for analysis using the “WGCNA” package (version 1.70–3). Initially, sample clustering was performed to eliminate outliers and to enhance the reliability of the findings. Subsequently, a trait heat map and sample dendrogram were created and an optimal soft threshold was established. Data were segmented into modules using a dynamic tree-cutting technique. Modules demonstrating the strongest correlations with the ssGSEA scores of NETRGs were selected for further investigation.

### 2.4. Functional enrichment analysis

Candidate genes were identified by overlapping DEGs with key module genes associated with ssGSEA scores for NETRGs. Enrichment analysis for these candidate genes, using gene ontology and Kyoto encyclopedia of genes and genomes (KEGG), was applied using “clusterProfiler” package,^[21]^ adopting a threshold of p.adj < .05.

### 2.5. Machine learning

For the GSE40888 dataset, Support vector machine recursive feature elimination (SVM-RFE) and least absolute shrinkage and selection operator algorithms were employed to screen important genes. Biomarkers were then identified by intersecting the genes highlighted by both the machine-learning techniques. Correlations between biomarkers were calculated using Pearson algorithm.

### 2.6. Immune feature and gene set enrichment analysis

The ssGSEA algorithm was used to assess the abundance of 28 immune cells within CA samples.^[22]^ Next, correlation between diagnostic biomarkers and differential immune cells were counted and presented. Additionally, GSEA was used to probe the potential KEGG pathways linked to biomarkers via clusterProfiler’^[23]^ with *P*.adj < .05.

### 2.7. Construction of regulatory network

Transcription factors related to biomarkers were predicted using the NetworkAnalyst database (https://www.networkanalyst.ca/). Meanwhile, miRNAs linked to biomarkers were predicted with mirDIP (http://ophid.utoronto.ca/mirDIP/) and miRWalk databases (http://mirwalk.umm.uni-heidelberg.de/). “TF-mRNA” and “mRNA-miRNA” network were constructed via Cytoscape.^[24]^

### 2.8. Expression of biomarkers

To validate the findings from the analysis of the public database, 10 paired control and CA samples were obtained from clinical sources for RNA extraction and RT-qPCR assays.The study was approved by the Ethics Committee of General Hospital of Ningxia Medical University (number: KYLL-20241032). The study was conducted in accordance with the local legislation and institutional requirements.This study obtained informed consent from all participating patients. Total RNA from these 20 whole blood samples was extracted using TRIzol (Ambion, Austin, TX, USA) following the manufacturer’s instructions. Reverse transcription of the RNA into cDNA was conducted using a First-strand-cDNA-synthesis-kit (Servicebio, Wuhan, China) according to the manufacturer’s protocol. Subsequent qPCR analysis was performed using 2 × Universal Blue SYBR Green qPCR Master Mix (Servicebio, Wuhan, China), according to the provided guidelines. The primers used for the PCR are listed in Table 1. Gene expression levels were normalized to the internal control GAPDH and calculated using the 2^−ΔΔCt^ method.^[25]^

**Table 1:** List of RT-qPCR primer sequences.

Genes	Primers
FCGR2B F	CGCTGTACTCATCCAAGCCT
FCGR2B R	GGAACCTCTGCACCAACGAG
FCRL5 F	ATTGTCATCAGCCTGGTGGG
FCRL5 R	GGTGTCAAGTGCCGACCTTA
CCR2 F	GGCATAGGGCAGTGAGAGTC
CCR2 R	CGCTTGGTGATGTGCTTTCG
FCRL1 F	GTGACCCTGACGTGTAAGATG
FCRL1 R	TCGCACCAGTATGACCCTGT
GAPDH F	CCCATCACCATCTTCCAGG
GAPDH R	CATCACGCCACAGTTTCCC

### 2.9. Statistical analysis

All P-values of the statistical results were based on 2-sided statistical tests, and a *P* value < .05 was considered statistically significant.

## 3. Results

### 3.1. Identification of 2611 key module genes in CA

**Figure 1.**
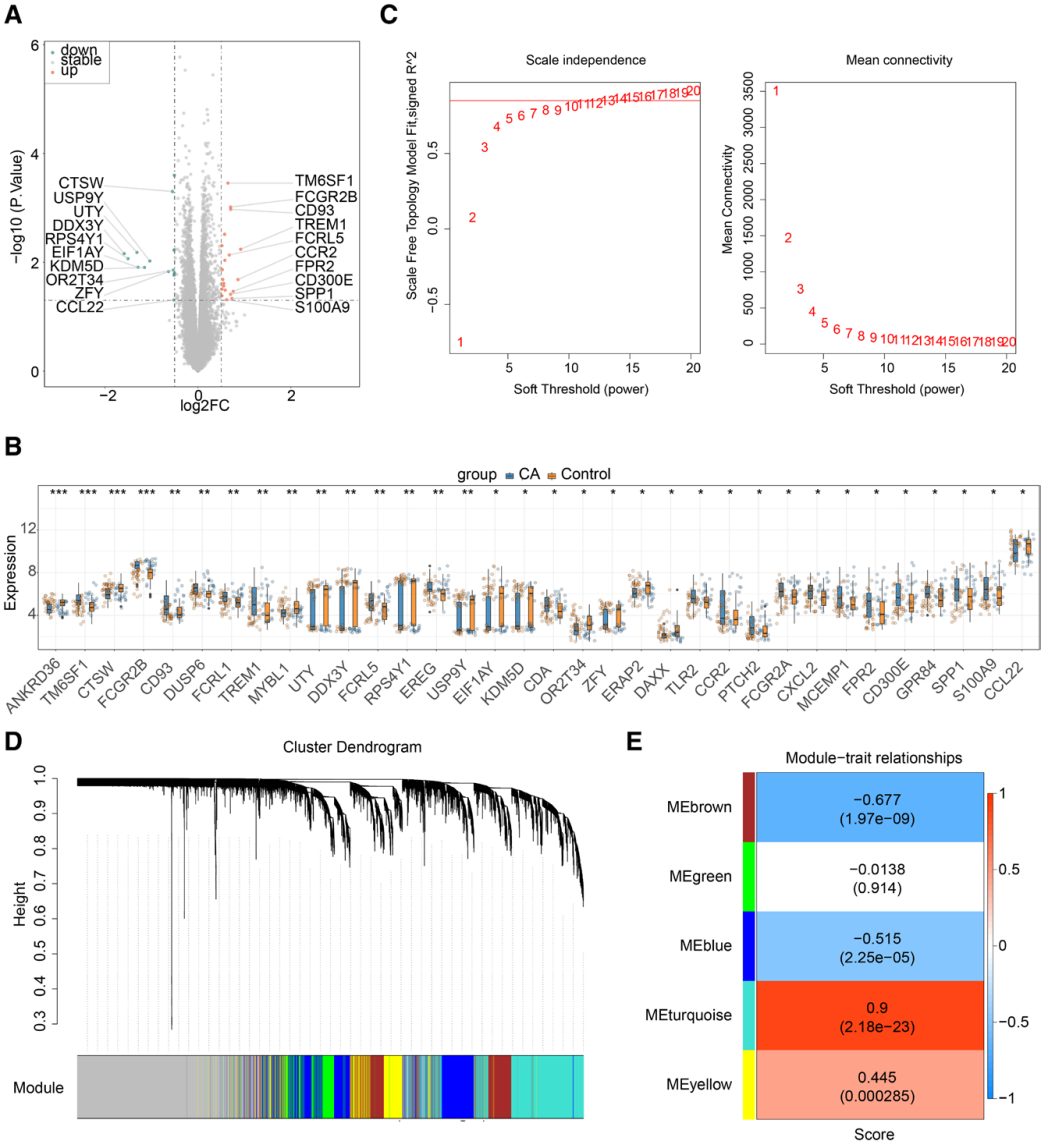
Identification of DEGs and module genes (A) Volcano map of DEGs between the normal and CA groups in the GSE40888. (B) Expression of DEGs between the normal and CA groups in the GSE40888 (C) Scale-free fit indices and average connectivity for soft threshold (β) (D) Gene modules identified by WGCNA (E) Correlation of gene modules with ssGSEA scores. CA = childhood asthma, DEGs = differentially expressed genes, WGCNA = weighted gene co-expression network analysis.

Altogether, 34 DEGs were identified in CA, comprising 14 downregulated and 20 up-regulated genes (Fig. 1A and B). To identify the key modules associated with the ssGSEA scores for NETRGs, WGCNA was conducted. This analysis confirmed the absence of outlier samples, and determined an optimal soft threshold of 14. The scale-free fit index, depicted as the signed R^2^ value, approached the desired threshold of 0.85 (illustrated by the red line), indicating a mean connectivity near zero (Fig. 1C). Subsequently, 6 modules were identified (Fig. 1D), among which the MEturquoise module showed a significant correlation with ssGSEA scores for NETRGs (correlation = 0.9, *P* < .05) (Fig. 1E). Thus, analysis of the MEturquoise module yielded 2611 genes for further investigation.

### 3.2. Functional enrichment analysis of 11 candidate genes

**Figure 2.**
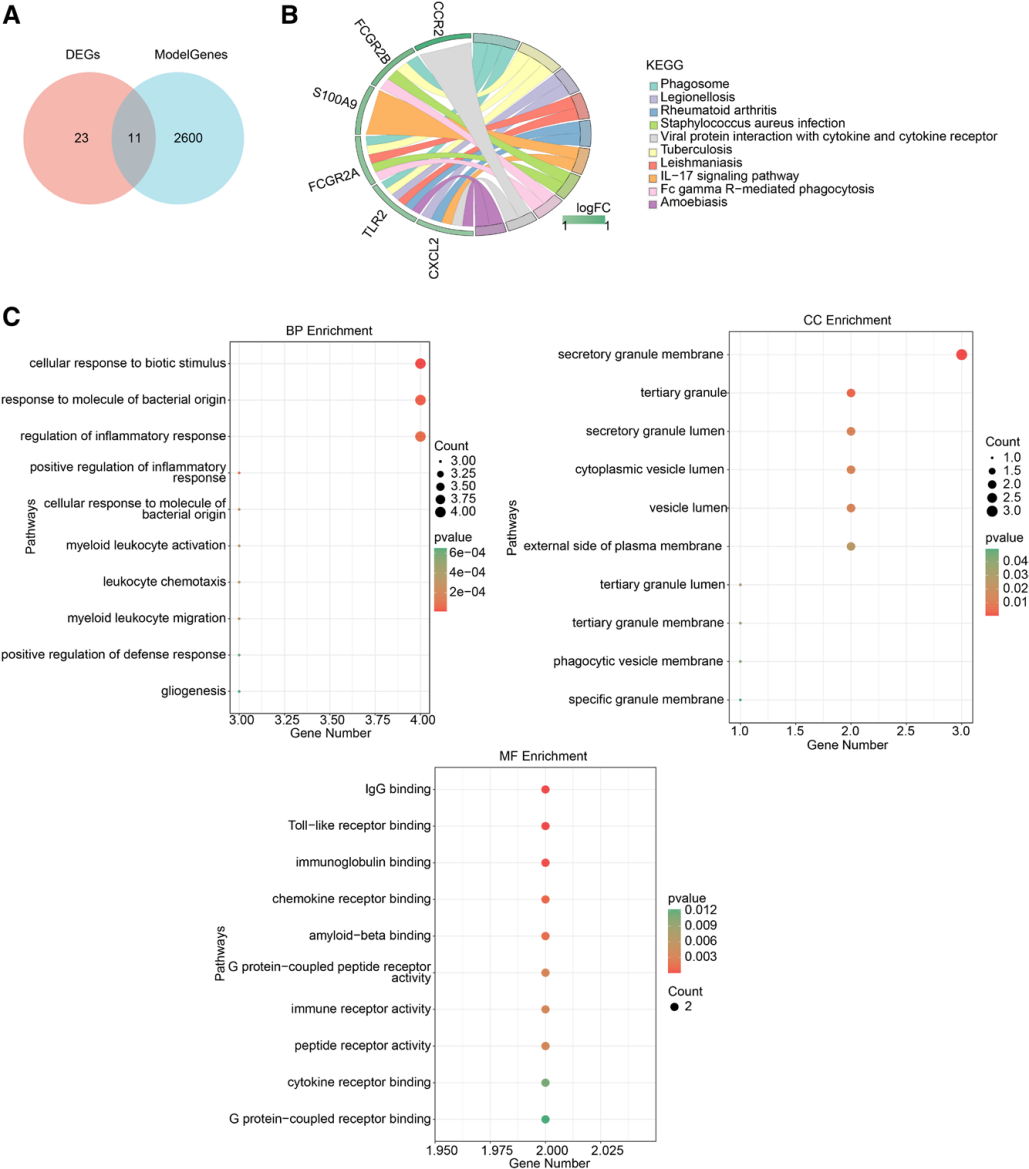
Functional enrichment analysis of candidate genes. (A) Candidate genes by intersecting DEGs with module genes. (B) KEGG results of candidate genes. (C) GO results of candidate genes, including BP, CC, and MF. DEGs = differentially expressed genes, GO = gene ontology, KEGG = Kyoto encyclopedia of genes and genomes.

Subsequently, 11 candidate genes were identified by intersecting the set of 34 DEGs with 2611 key modules genes (Fig. 2A). Functional enrichment analysis was performed to uncover the possible roles of these candidate genes in connection with NETs. KEGG results suggested that these candidate genes were mainly enriched in viral protein interactions with cytokine and cytokine receptors and the IL-17 signaling pathway (Fig. 2B). In addition, the top 10 gene ontology terms under each item are displayed in Figure 2C, showing that these genes were associated with “positive regulation of defense response” and “regulation of inflammatory response.”

### 3.3. FCGR2B, FCRL5, CCR2, and FCRL1 were identified as biomarkers for CA

**Figure 3.**
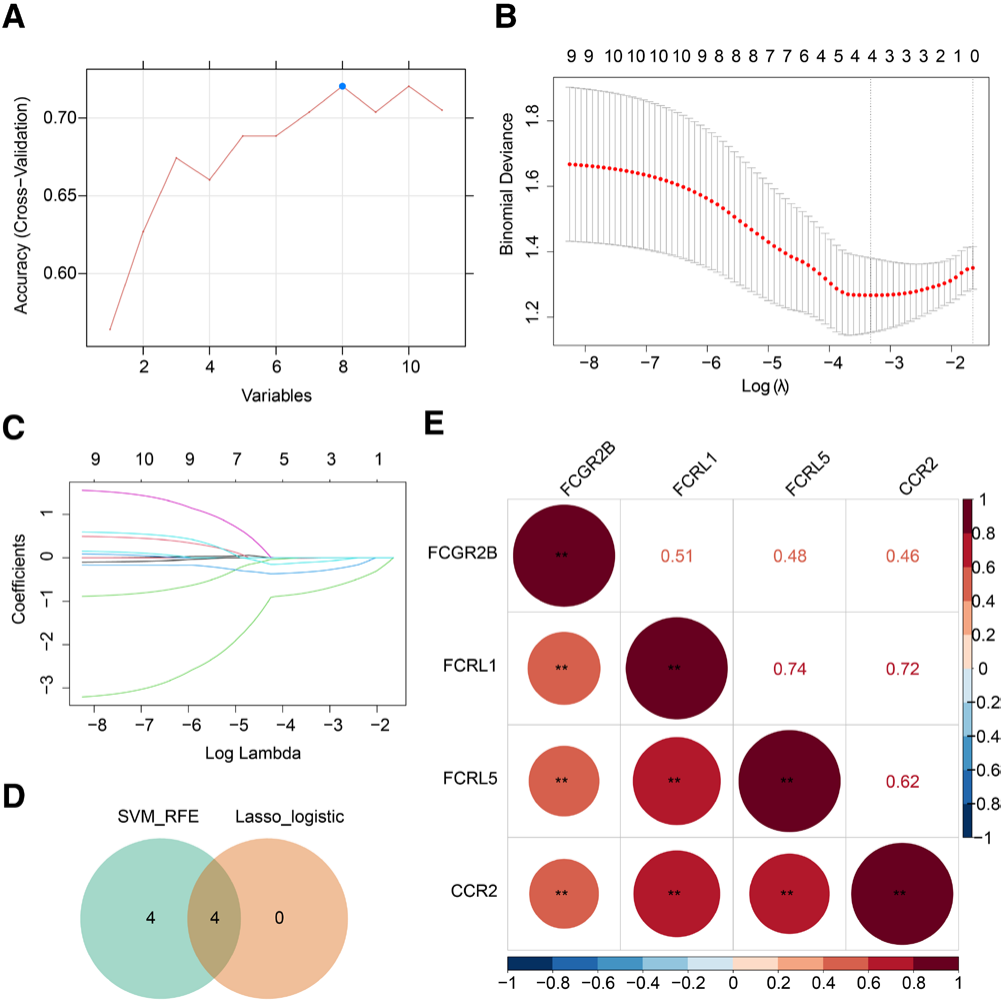
Screening biomarkers by machine learning. (A) Accuracy curves for SVM-RFE. (B) Gene coefficient for LASSO. (C) Cross-validation error plot for LASSO. (D) Four biomarkers were produced by overlapping feature genes from SVM-RFE and LASSO. (E) Correlation between biomarkers. LASSO = least absolute shrinkage and selection operator, SVM-RFE = support vector machine recursive feature elimination.

To refine the identification of key genes, SVM-RFE analysis was carried out on 11 candidate genes, resulting in the selection of 8 feature genes: FCGR2B, FCRL5, CCR2, FCRL1, GPR84, S100A9, CXCL2, and FCGR2A (Fig. 3A). Meanwhile, 4 feature genes were produced by the least absolute shrinkage and selection operator-logistic algorithm, including CCR2, FCRL5, FCGR2B, and FCRL1 (Fig. 3B and C). These 4 genes were subsequently designated as NET-related biomarkers in CA (Fig. 3D). Therefore, these genes have been defined as neutrophil extracellular trap-related biomarkers in CA. The correlation between the 4 biomarkers was positive, and the correlation between FCRL1 and FCRL5 was the strongest (Fig. 3E).

### 3.4. Immune infiltration and function analysis of biomarkers

**Figure 4.**
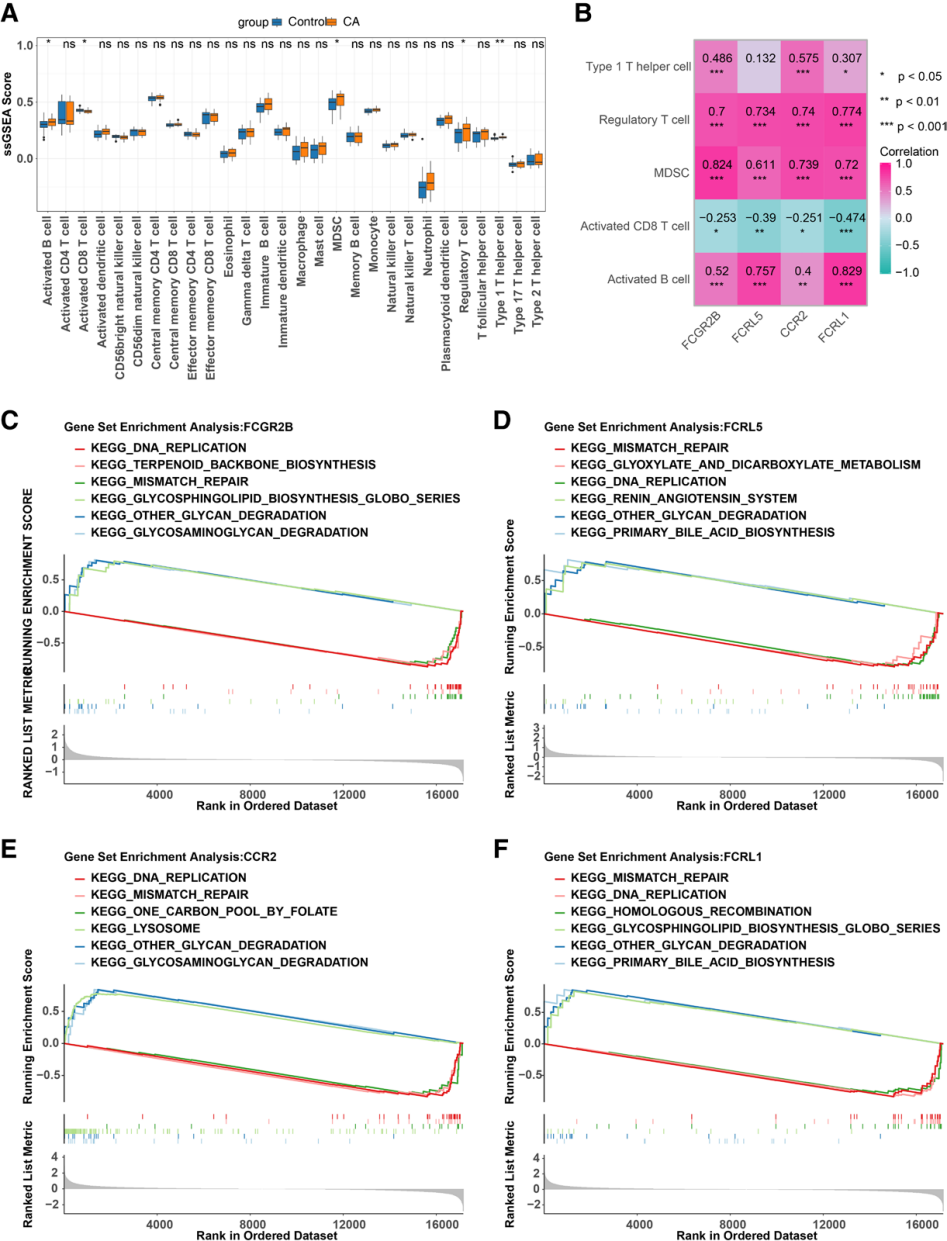
Immune infiltration analysis and function of biomarkers. (A) Expression abundance of 28 immune cells between the normal and CA groups in the GSE40888. (B) Correlation of biomarkers with differential immune cells. KEGG pathway for FCGR2B (C), FCRL5 (D), CCR2 (E), and FCRL1 (F).

To investigate the immune microenvironment of CA, we analyzed the abundance of 28 immune cells in the 2 groups from the GSE40888 dataset. We found significant differences in the abundance of 5 types of immune cells: activated B cells, activated CD8 T cells, myeloid-derived suppressor cells (MDSC), regulatory T cells, and type 1 T helper cells (Fig. 4A). Correlation analysis indicated a negative association between activated CD8 T cells and all identified biomarkers. In contrast, activated B cells, MDSC, regulatory T cells, and type 1 T helper cells positively correlated with these biomarkers (Fig. 4B). Further exploration of the roles of FCGR2B, FCRL5, CCR2, and FCRL1 in CA using single-gene GSEA revealed that “other glycan degradation” pathways were related to the high expression of FCGR2B, FCRL5, CCR2, and FCRL1 (Fig. 4C–F).

### 3.5. Analysis of the interaction regulation of biomarkers

**Figure 5.**
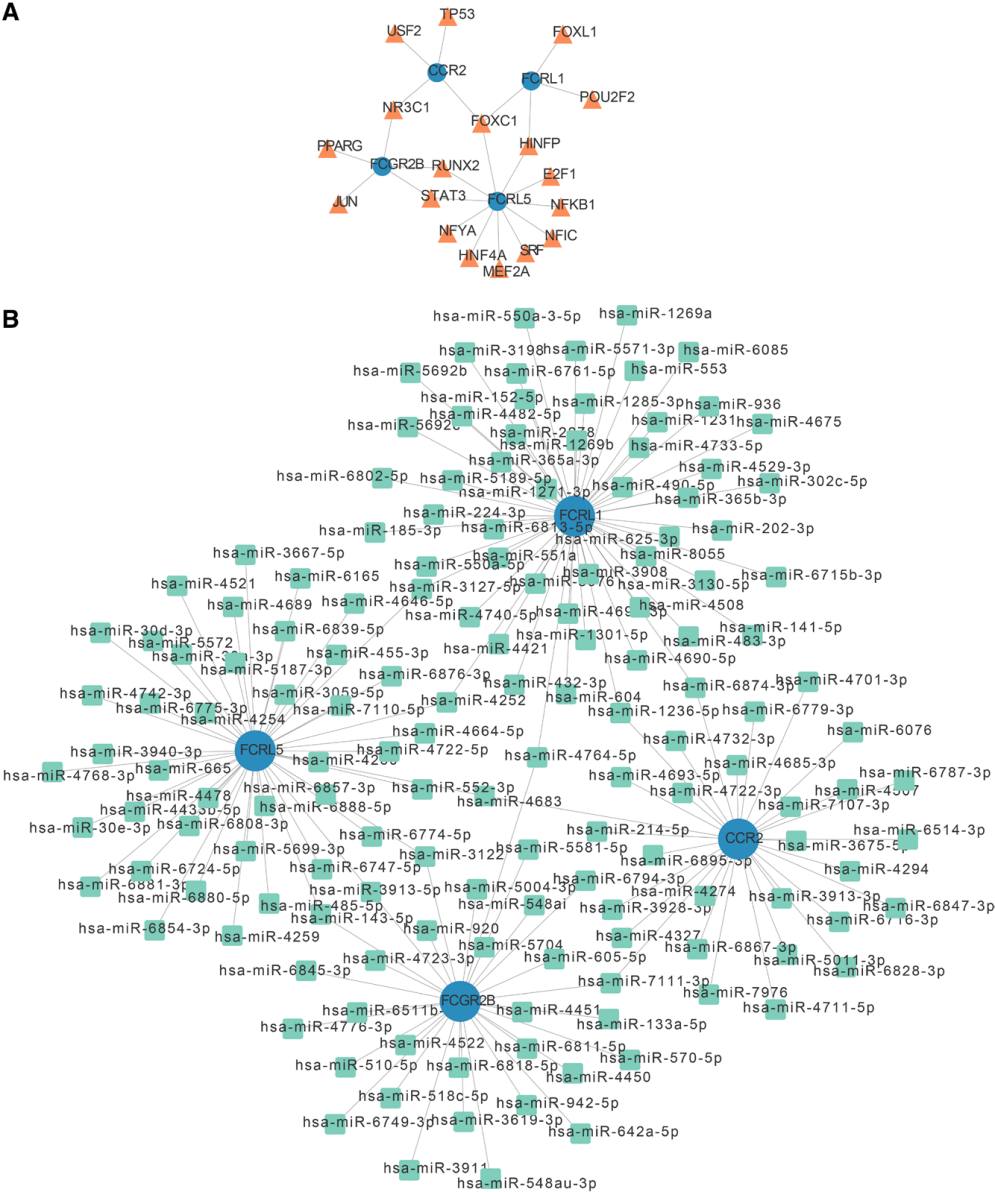
Molecular regulatory networks of biomarkers (A) TF-mRNA network. (B) The mRNA-miRNA network. miRNA = microRNA, TF = transcription factor.

To delve into regulatory interactions, networks for “TF-mRNA” and “mRNA-miRNA” were developed. FOXC1 regulated FCRL5, CCR2, and FCRL1 expression (Fig. 5A). The network involving “mRNA-miRNA” network contained 153 nodes and 159 edges, with hsa-miR-3127-5p simultaneously linked to FCRL5 and FCRL1 (Fig. 5B).

### 3.6. Expression validation of the biomarkers

**Figure 6.**
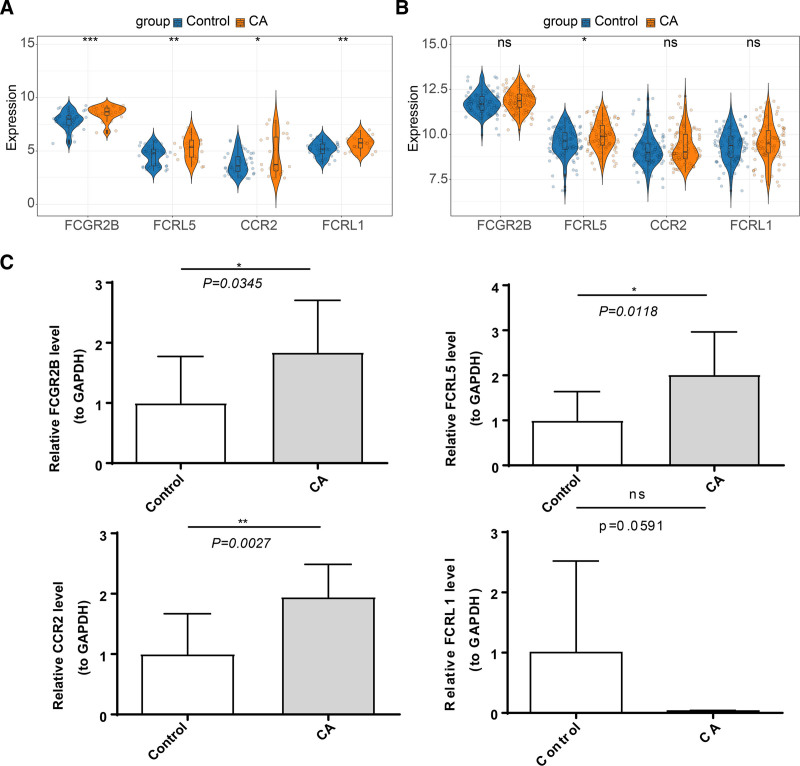
Biomarker expression profile. (A) Biomarker expression profile in GSE40888 dataset. (B) Biomarker expression profile in GSE40732. (C) Biomarker expression profile by RT-qPCR. *P < .05, **P < .01, ***P < .001, ****P < .0001. ns = nonsignificance.

In the analysis of biomarkers at the transcriptome level from datasets GSE40888 and GSE40732, we noted elevated expression of FCGR2B, FCRL5, CCR2, and FCRL1 in the CA group relative to the normal group (Fig. 6A and B). These findings were further confirmed by RT-qPCR of clinical blood samples. These data concurred with our initial analyses, showing significantly higher levels of FCGR2B, FCRL5, and CCR2 in the CA group than in the normal group (Fig. 6C). Nonetheless, FCRL1 expression showed a contrasting trend, likely due to sample heterogeneity.

## 4. Discussion

Asthma is a prevalent chronic respiratory condition that poses a significant threat to children’s health.^[26,27]^ The number of asthma patients is still increasing, especially in developed countries.^[28]^ Given the current situation both domestically and internationally, as well as recent studies have shown an association between NETs and various diseases such as asthma,^[29]^ acute lung injury,^[30]^ cystic Fibrosis,^[31]^ systemic lupus erythematosus^[32]^ and cancer.^[33]^ Because of the effects of NETs in asthma, we tried to find the identified pathways in children asthma (CA).

In this study, 11 candidate genes were identified by taking the intersection of 34 DEGs and 2611 key module genes. Using machine-learning algorithms, *FCGR2B*, *FCRL5*, *CCR2*, and *FCRL1* have been identified as NET-related biomarkers in CA. After validation by RT-qPCR, only the expression of *FCGR2B*, *FCRL5*, and *CCR2* was consistent at the transcriptome level.

Encoded by the Fc gamma receptor IIB (*FCGR2B*) gene, the FCGR2B protein serves as a crucial immune receptor that is primarily located on the surface of immune cells.^[34]^ This receptor plays a crucial role in regulating the activation and function of immune cells. Specifically, *FCGR2B* modulates the signaling of immune cells by binding to antibodies, affecting cell activation, proliferation, secretion, and other functions, thereby influencing the intensity and type of immune response.^[35]^ Additionally, *FCGR2B* is involved in regulating the self-tolerance of immune cells, helping to prevent the immune system from attacking its own tissues and maintaining immune balance.^[34]^ Therefore, the *FCGR2B* and its encoded protein play a significant role in immune regulation, contributing to the maintenance of immune function and prevention of autoimmune diseases.^[36,37]^ Deficiency in *FCGR2B* accelerates arthritis progression and surpasses the protective effects offered by a lack of complement C5.^[35]^

Fc receptor-like 5 (*FCRL5*) encodes a protein that belongs to the Fc receptor-like (FCRL) family. *FCRL5*, which is mainly found in B cells, influences their signaling and functional capabilities. It is involved in the control of B cell activation, growth, and the production of antibodies.^[38]^ Through interactions with antibodies, *FCRL5* potentially affects the immune response by setting the activation thresholds for B cells. Studies have suggested that *FCRL5* may be involved in autoimmune diseases, allergic reactions, and other immune-related disorders. Understanding the function of *FCRL5* can provide insights into the mechanisms underlying these conditions, and may lead to the development of targeted therapies. FCRL proteins are a group of receptors that share structural similarities with classical Fc receptors but have distinct functions in immune regulation^[39]^ surface of B cells and plasma cells, with higher expression observed in plasma cells of patients with multiple myeloma. This makes it a potential target for ADCs (antibody–drug conjugates) in multiple myeloma treatment.^[38]^ Our research has made a novel discovery, identifying the *FCRL5* gene as a potential diagnostic biomarker for cancer diagnosis.

CC chemokine receptor 2 (*CCR2*) is a key receptor for monocyte migration, making it a potential target for therapeutic intervention in autoimmune diseases, atherosclerosis, pain, and metabolic disorders.^[40]^ A recent study explored the elevated expression of the *CCL2*/*CCR2* axis in the bronchial smooth muscles in allergen-challenged mice, suggesting that it is a possible factor for airway hyperresponsiveness in asthma.^[41]^ Other study results indicate that targeting a single chemokine receptor in the early stages of asthma development can impact the disease later, highlighting the potential efficacy of anti-*CCR2* monoclonal antibodies in asthma treatment.^[42]^ Blocking the *CCL2*/*CCR2* signaling pathway leads to a reduction in allergic asthma biomarkers (such as eosinophils), suppression of Th2 inflammatory responses, and enhancement of Th1-related cytokines.^[43]^ Besides, due to the importance of *CCR2* and 4-azetidinyl-1-heteroatom linked cyclohexane antagonists, which could provide a new target for asthma therapy.^[40,44]^

GSEA was used to investigate pathways related to the biomarkers. These genes are involved in several important pathways, including mismatch repair/DNA replication/homologous recombination and other glycan degradation pathways. Ferroptosis is another crucial pathway to children’s asthma, either.Ferroptosis is another crucial pathway to children’s asthma, either.^[45]^ For example, Jehan Alladina and colleagues identified dynamic changes and up-regulated of genes in the asthmatic airway epithelium, and identified specific cell populations, such as pathogenic Th2 cells expressing IL9, conventional type 2 dendritic cells (DC2), and monocyte-derived cells expressing *CCR2*, which were uniquely enriched in asthmatic patients following allergen challenge.^[46]^ Susana Rojo-Tolosa and colleagues found that certain polymorphisms of genes such as *FCER1A*, *FCGR2B*, and *GATA2* were associated with different aspects of treatment response, including reduced exacerbations, improved lung function, and decreased use of oral corticosteroids,^[47]^ it’s the same as children’s asthma.

Furthermore, the mechanism of asthma is related to several cell types and cytokines, including neutrophils, type 1 T helper cells (Th1), innate lymphoid type 2 cells (ILC2s), Th7 cells, and IL-6 and IL-17, as these inflammatory cells and cytokines infiltrate, leading to airway obstruction and spasms.^[14,17,48]^ In our study, we found a significant relationship between these biomarkers and inflammatory cells, including activated B cells, activated CD8 T cells, MDSC, regulatory T cells, and Th1 cells, which showed significant differences (*P* < .05). The relationship between these cells and biomarkers is not entirely positive, and some studies have proven this before.^[49–52]^ This illustrates that different inflammatory cells have a variety of effects in different pathways.^[53–55]^

Several studies have indicated that various lncRNAs and mRNAs could play a direct role in the development of CA by interacting with the ECM receptor.^[1,15,56]^ By predicting the transcription factors and miRNAs of biomarkers, the factors involved in the transcriptional regulation of biomarkers were analyzed, and a molecular network regulating biomarkers was constructed, providing a reference for subsequent experiments.

Based on this research, these findings could help us to identify potential biomarkers linking neutrophil-related genes in CA disease states to immune,^[20,57]^ molecular pathways, and transcriptional regulatory networks, providing insights into the study of NETRGs in CA diseases.^[2,58,59]^ Better treatments exclude inhaled corticosteroids and antihistamines to cure asthma.^[60,61]^ However, we did not perform more in-depth experiments to verify the expression of these genes, so we could perform western blotting or flow cytometry if we had adequate experimental conditions. In conclusion, defining a deeper mechanism of asthma is still difficult. There is a long way to explore this issue. Future research should break the barrier between basic and clinical medicine, and elucidate a clearer and more accurate pathogenesis.

In conclusion, our study identified 34 differentially expressed genes (DEGs) associated with CA and 4 key biomarkers (FCGR2B, FCRL5, CCR2, and FCRL1) linked to NETs in the context of CA. Using advanced machine-learning techniques and immune infiltration analysis, we also observed differential immune cell infiltration in the CA immune microenvironment. Furthermore, all identified biomarkers were associated with the “other glycan degradation” pathways and exhibited significantly higher expression levels in the CA group than in the control group. These findings provide a theoretical basis for the development of treatment strategies for CA.

## Acknowledgments

We would like to express our sincere gratitude to all individuals and organizations who supported and assisted us throughout this research. In conclusion, we extend our thanks to everyone who supported and assisted us in this way. Without your support, this study would not have been possible.

## Author contributions

**Conceptualization:** Yuexuan Wu.

**Data curation:** Wen Zhao.

**Formal analysis:** Yalong Yang.

**Methodology:** Wen Zhao.

**Project administration:** Yuexuan Wu.

**Visualization:** Jinhai Ma.

**Writing – original draft:** Yuexuan Wu.
